# Objections to Compulsory Notification of Phthisis

**Published:** 1910-06-11

**Authors:** 


### Medicine.

### OBJECTIONS TO COMPULSORY NOTIFICATION OF PHTHISIS.

It is by no means every specialist in tuberculous affections, nor even every medical man who specialises in sanatorium treatment, that is an advocate of the compulsory notification of phthisis, and some of the arguments against such compulsory notification have been well summarised by Dr. Muthu in a book of his that we have recently reviewed.* He points out that pulmonary tuberculosis is essentially a chronic disease in the great majority of cases, and that it can by no means be regarded as "a dangerous infectious disorder."

The word "contagion" should not be used in regard to consumption, as it is not a contagious disease in the same sense as glanders or syphilis. It is the want of a better understanding of the character and behaviour of the disease that has led medical men to join hands with some lay members of the community to demand its compulsory notification under the Public Health Acts. It is well to point out the objections against taking such a serious step.

Tuberculosis possesses a low degree of communicability, and if ordinary precautions are taken to insure proper ventilation and the disposal of the sputum, the infection will be almost nil.

When we come to consider who is tuberculous and who is not, serious difficulty at once begins. Almost everyone living in any town is potentially tuberculous, and carries more or less some tubercle germs with him. Hundreds of cases that are not recognised or treated in life are spontaneously cured. If the disease is to be recognised by its early signs, the general practitioner may not be conversant with what the specialist would consider early physical indications; and if the specialists themselves differ, who is to decide that a patient is suffering from consumption?

If the presence of the tubercle bacillus is to decide the fate of the patient, then we are confronted with fresh difficulties. A patient may expectorate one day and not the next or the following days. Dr. Muthu had a patient who stopped expectorating for six months, and then brought a little just once, although he had well-marked disease all the time. In cases of a late stage of the disease, in cases of cavity, or where fibrosis is most marked, patients do not always bring up sputum. Even when there is expectoration, tubercle bacilli may be found one day, and the next day they may either be absent or not demonstrable. Two medical men examining the sputum on these two occasions will come to opposite conclusions.

If the advocates of the Act think that by making notification compulsory they would be able to deal with early cases, they are much mistaken; they cannot know human nature. The effect of the Act would be for patients to conceal their disease. They will not face the ordeal of being examined, if such examination lets loose the terrors of boycott and ostracism. Some panic-mongers have kept up an ignorant agitation, frightening people into thinking that consumption is a highly contagious disease, and that every consumptive is a hotbed of infection to be avoided at all cost. The consequence of this absurd scare of infection must be that the public will shun the consumptive as if he were a leper or a pariah. It is bad enough now to get patients in the early stage for treatment. The loss of time and money, besides the inconvenience of leaving home and work, are so great that patients put off consulting a medical man or taking a decisive step for a long time; and if a Compulsory Notification Act were passed it would give the patients an additional encouragement to suppress their disease longer still till it might be too late for successful treatment.

To prove that this is true in actual practice, Dr. Muthu cites an instance in his own experience. A young clerk went back to his office after being in a sanatorium for seven months, his disease having been entirely arrested. The morning he presented himself at the office his fellow-clerks consulted together, went to their chief and petitioned him not to take this patient back, and that if he did they would all resign. The consequence was that he lost his situation, although, as he said, he was in every way much healthier and stronger than any of his companions at the desk. It so happened that one of his fellow-clerks was himself a consumptive, and had already made arrangements to go to a santorium to be treated. But the attitude of the others, and the treatment the patient had received at their hands, so terrified him that he abandoned all idea of leaving his place, and dragged on four more months, working away as if nothing was wrong with him. When he became too ill to work any longer he went home, took to bed, and died within a month. If compulsory notification were to become law, such cases would increase and add to the suffering of the consumptive who, besides being very sensitive, is already handicapped in life with a mortal disease.

Those who press for compulsion with the idea that it would help consumptive cases to be isolated, and thus stamp out the spread of the disease, forget that it is a moot question whether segregation would diminish the incidence of tuberculosis. Dr. James, in a paper he read before the Edinburgh Medico-Chirurgical Society, drew attention to certain curves showing the mortality from phthisis per 10,000 of the population between the years 1860 and 1905 in Aberdeen, Dundee, and Edinburgh. Here there had been only the progressive voluntary efforts of individuals to improve the general conditions of life, and the results were as satisfactory as they were distinct. Another set of curves related to typhoid fever during the same period and in the same towns. The enforcement of legislative enactments did not appear to have improved matters as regards the disease. As regards measles, the curves showed that since notification and isolation the mortality had all over increased rather than diminished.

* "Pulmonary Tuberculosis and Sanatorium Treatment."

Dr. Killick Millard, in an article on the "Influence of Hospital Isolation for Scarlet Fever," says "hospital isolation of scarlet fever in towns appeared to have failed to reduce materially the prevalence of the disease as well as its fatality." Besides the ratio of scarlet fever cases (in the jurisdiction of the Metropolitan Asylums Board) to population had increased from 3.7 per 1,000 in 1890 to 4.3 in 1906. If such be the case in regard to typhoid fever, measles, and scarlet fever, how can we expect that mere isolation would prevent the spread or cause the fall of the death rate of tuberculosis? Dr. James Wheatley, the medical officer for Salop, is driven to the conclusion that isolation of phthisis in asylums and workhouses has not been a prominent factor in the decrease of phthisis in Shropshire during the last fifty years. Besides, one should remember that the death rate from pulmonary tuberculosis declined long before the introduction of notification and has fallen in other places which have not notified in like or even greater amount, in the absence of any direct measures whatever.

The adoption of some such system as voluntary notification might prove useful, in that it would lead to preventive and educational measures being taken in insanitary areas, or to the removal from crowded tenements and unhygienic conditions of the consumptive poor who have no one to look after them, or who may be in danger of infecting other inmates of the same room. But even the voluntary notification by the general practitioner presents dangers and risks when looked at, not only from a clinical and ethical but also from a legal point of view. For, apart from the difficulties of rightly diagnosing an early case of consumption, and apart from the objection to the notification, for reasons given above, the medical man would be liable to incur serious damages if he wrongly notified a case or if his diagnosis, even though right, were not supported by other expert medical evidence. If a highly contagious disease like syphilis is not notified, it is not possible to bring consumption under the notification Act, especially when it is regarded as possessing only a low degree of infection.

